# Developmental Neurotoxicity of Fipronil and Rotenone on a Human Neuronal In Vitro Test System

**DOI:** 10.1007/s12640-021-00364-8

**Published:** 2021-04-19

**Authors:** Anne Schmitz, Silke Dempewolf, Saime Tan, Gerd Bicker, Michael Stern

**Affiliations:** Institute of Physiology and Cell Biology, University of Veterinary Medicine Hannover, Bischofsholer Damm 15/102, 30173 Hannover, Germany

**Keywords:** DNT, Differentiation, Migration, Neurite outgrowth, NTera-2, NT2

## Abstract

Pesticide exposure during in utero and early postnatal development can cause a wide range of neurological defects. However, relatively few insecticides have been recognized as developmental neurotoxicants, so far. Recently, discovery of the insecticide, fipronil, in chicken eggs has raised public concern. The status of fipronil as a potential developmental neurotoxicant is still under debate. Whereas several in vivo and in vitro studies suggest specific toxicity, other in vitro studies could not confirm this concern. Here, we tested fipronil and its main metabolic product, fipronil sulfone both at concentrations between 1.98 and 62.5 µM, alongside with the established developmental neurotoxicant, rotenone (0.004–10 µM) in vitro on the human neuronal precursor cell line NT2. We found that rotenone impaired all three tested DNT endpoints, neurite outgrowth, neuronal differentiation, and precursor cell migration in a dose-dependent manner and clearly separable from general cytotoxicity in the nanomolar range. Fipronil and fipronil sulfone specifically inhibited cell migration and neuronal differentiation, but not neurite outgrowth in the micromolar range. The rho-kinase inhibitor Y-27632 counteracted inhibition of migration for all three compounds (EC50 between 12 and 50 µM). The antioxidant, n-acetyl cysteine, could ameliorate the inhibitory effects of fipronil on all three tested endpoints (EC 50 between 84 and 164 µM), indicating the involvement of oxidative stress. Fipronil sulfone had a stronger effect than fipronil, confirming the importance to test metabolic products alongside original pesticides. We conclude that in vitro fipronil and fipronil sulfone display specific developmental neurotoxicity on developing human model neurons.

**Supplementary Information:**

The online version contains supplementary material available at 10.1007/s12640-021-00364-8.

## Introduction

In our daily life, a wide range of activities in agriculture, industry, and even the management of the own households is associated with using a diversity of chemicals, including pesticides (Schäfer et al. 2011). Insecticides do not only accumulate within the environmental circulation (Park et al. 2020); they are also absorbed via dietary intake or indoor and outdoor dust (Kim et al. 2019; Mahler et al. 2009). In addition to acute toxicity of high dose exposure, there are concerns about low-dose, long-term effects on human health. The developing fetal human brain is particularly vulnerable to chemical disturbance. Thus, developmental toxicity (DNT) of chemical compounds is an important issue that is given increasing attention. Several insecticides have been identified to cause DNT. The organophosphorous insecticide chlorpyrifos has been implicated as developmental neurotoxicant both in vivo and in vitro (Rauh et al. 2012; Slotkin and Seidler 2012). The developmental neurotoxic potential of pyretroids has recently been demonstrated on SH-SY5Y cells (Martinez et al. 2020). The pesticide rotenone, a mitochondrial electron transport chain blocker, is known for its adverse effect on dopaminergic neurons (Betarbet et al. 2000; Pamies et al. 2018), and is thus often used in Parkinson disease research (Heinz et al. 2017). However, it is also a selective inhibitor of axonal outgrowth of human neurons in vitro (Krug et al. 2013; Ryan et al. 2016). Recently, residues of the phenylpyrazole insecticide fipronil have been found in eggs in Europe, which led to public concerns about possible adverse effects on humans and to a significant attention in the daily press (e.g., BBC 2017; Zeit 2018).

The DNT status of fipronil is not yet finally agreed on. Several in vivo studies describe neurotoxic effects of fipronil in zebrafish development (Stehr et al. 2006; Park et al. 2020), rats (Cravedi et al. 2013; Abdel-Daim et al. 2019) and mice (Badgujar et al. 2016). In vivo testing of chemicals in vertebrates is cost-intensive and raises severe ethical concerns (Russell and Burch, 1959). Moreover, species differences preclude direct applicability of results obtained from animal experiments to the human central nervous system (Dragunow 2008; Leist and Hartung 2013). Alternatively, several in vitro systems have been developed in the past decade based on human cells in culture (e.g., Fritsche et al. 2018; Pamies et al. 2018; Pistollato et al. 2017; Krug et al. 2013; Ryan et al. 2016; Stern et al. 2014). A few studies have tested fipronil in vitro and found indications for a DNT potential (Lassiter et al. 2009; Sidiropoulou et al. 2011; Slotkin et al. 2016; Ruangjaroon et al. 2017), but not all of them show clear-cut results. In a study on neurite outgrowth of Lund human mesencephalic cells (LUHMES) by Krug et al. (2013), fipronil appeared unspecifically toxic. Based on this study, fipronil has been suggested as a DNT negative tool compound for the design of in vitro DNT assays (Aschner et al. 2017). Also, the mode of toxic action for fipronil is still not clear, although the involvement of reactive oxygen species is most likely involved (Wang et al. 2016).

In an attempt to clarify the situation, we here tested fipronil alongside with a clear DNT positive pesticide, rotenone, and the metabolic product of fipronil, fipronil sulfone, for DNT on three different endpoints in the same cell line. We employed the human cell line Ntera2, clone D1 (NT2), an embryonic carcinoma cell line that is terminally differentiated into neurons by treatment with a single morphogen, retinoic acid (RA) (Andrews et al. 1984; Pleasure et al. 1992). Based on our previous studies (Stern et al. 2014; Roloff et al. 2015), we tested effects on cell migration, neuronal differentiation, and neurite outgrowth.

## Materials and Methods

### Chemicals

All chemicals (Table 1) were obtained from Merck, Darmstadt, Germany, unless stated otherwise. Rotenone, fipronil, fipronil sulfone, cytochalasin D, and α-tocotrienol (a kind gift from the Institute for Food Toxicology, University of Veterinary Medicine Hannover) were dissolved in dimethyl-sulfoxide (DMSO) as stock solutions, resulting in maximally 0.25% DMSO in culture media. In pilot experiments, DMSO concentrations up to 1% had no observable adverse effect on our cell cultures. Sodium valproate, Y-27632, and n-acetyl-cysteine (NAC) were dissolved directly in cell culture media.

**Table 1:** Chemicals

Name	IUPAC name	CAS-Nr	Formula
Fipronil	5-Amino-1-[2,6-dichloro-4-(trifluoromethyl)phenyl]-4-(trifluoromethylsulfinyl)pyrazole-3-carbonitrile	120,068–37-3	C_12_H_4_Cl_2_F_6_N_4_OS
Fipronil sulfone	5-Amino-1-[2,6-dichloro-4-(trifluoromethyl)phenyl]-4-(trifluoromethylsulfonyl)pyrazole-3-carbonitrile	120,068–36-2	C_12_H_4_Cl_2_F_6_N_4_O_2_S
Rotenone	(1*S*,6*R*,13*S*)-16,17-Dimethoxy-6-prop-1-en-2-yl-2,7,20-trioxapentacyclo[11.8.0.0^3,11^.0^4,8^.0^14,19^]henicosa-3(11),4(8),9,14,16,18-hexaen-12-one	83–79-4	C_23_H_22_O_6_
Cytochalasin D	[(1*R*,2*R*,3*E*,5*R*,7*S*,9*E*,11*R*,12*S*,14*S*,15*R*,16*S*)-16-Benzyl-5,12-dihydroxy-5,7,14-trimethyl-13-methylidene-6,18-dioxo-17-azatricyclo[9.7.0.0^1,15^]octadeca-3,9-dien-2-yl] acetate	22,144–77-0	C_30_H_37_NO_6_
α-tocotrienol	(2*R*)-2,5,7,8-Tetramethyl-2-[(3*E*,7*E*)-4,8,12-trimethyltrideca-3,7,11-trienyl]-3,4-dihydrochromen-6-ol	58,864–81-6	C_29_H_44_O_2_
Y-27632	4-[(1*R*)-1-Aminoethyl]-*N*-pyridin-4-ylcyclohexane-1-carboxamide	146,986–50-7	C_14_H_21_N_3_O
NAC	(2*R*)-2-Acetamido-3-sulfanylpropanoic acid	616–91-1	C_5_H_9_NO_3_S
Sodium valproate	Sodium; 2-propylpentanoate	1069–66-5	C_8_H_15_NaO_2_
DMSO	Methylsulfinylmethane	67–68-5	C_2_H_6_OS

### Cell Culture

**Fig. 1:**
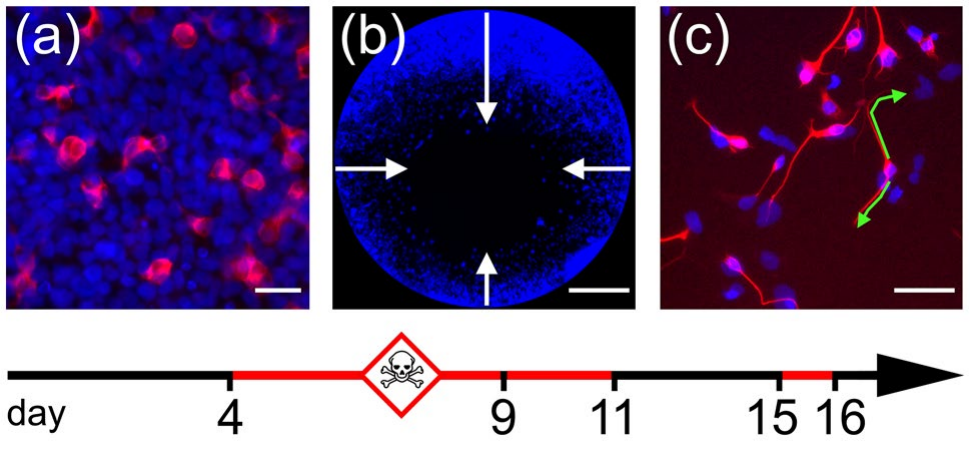
Three DNT endpoints assessed on NT2 cultures in vitro. (**a**) Neuronal differentiation was assessed by measuring immunofluorescence of a monoclonal antibody against b-tubulin type III. On average, ~ 10% of all DAPI-positive cells (blue) display ß-tub III-immunoreactivity (red) after 9 days in culture under control conditions. (**b**) NT2 precursor cell migration was measured using the Oris cell migration assay, which creates cell culture monolayers with a circular hole. During 2 days in culture, cells migrate a distance of ~ 400 µm on average into this hole. (**c**) Neurite growth was assessed by cultivating dissociated NT2 cultures after 2 weeks exposure to retinoic acid (containing ~ 20–40% neurons) and measuring ß-tubulin type III labeled neurites (red) after 24 h. Green lines: example measurements of two neurites, 46 µm and 76 µm long. Scale bars: 50 µm (**a**, **c**), 400 µm (**b**)

The human Ntera2/D1 cell line (NT2, RRID: CVCL_3407) was obtained from the American Type Culture Collection, VA, USA. Cells were maintained and cultivated in DMEM/F12 culture medium (Invitrogen, Darmstadt, Germany) supplemented with 10% fetal bovine serum (Invitrogen), and 1% penicillin/streptomycin (Invitrogen) in an atmosphere of 5% CO_2_ at 37 °C. Using a differentiation protocol in free-floating aggregates (Paquet-Durand et al. 2003), 95% pure postmitotic neurons could be generated within 28 days. Neuronal differentiation and precursor cell migration are measured in 96-well plates on days 9, 11, or 16, respectively (Fig. 1). Each plate contained a dilution series of seven concentrations with six technical replicates of the same concentration per experiment, and each experiment was performed three times on different passages of NT2 precursor cells. For each test compound, relevant concentrations were determined in a range finding experiment with log 10 dilutions (data not shown), before deciding on the final range of concentrations used.

### Neurite Outgrowth Assay

Similar to our previous study (Roloff et al. 2015), experiments were performed with NT2 cells treated for 2 weeks with retinoic acid (2wkRA) from passage 27 to 35. Dispersed cells were seeded into two identical poly-D-lysine (10 µg/ml) coated 96-well plates (Corning Costar, Kaiserslautern, Germany) at a density of 10,000 cells per well. Cultures were exposed to up to seven different concentrations of a test compound in DMEM/F12 (six technical replicates per concentration) for 24 h. One plate was immediately fixed in 4% paraformaldehyde in PBS (PFA) for 15 min. After 24 h, while a resazurin reduction assay (2 h) was performed on the other plate. This was necessary, because even completely inhibited neurites immediately started growing in the resazurin assay. Fixed cells were washed in PBS containing 0.1% Triton X100 (PBS-T) and immunolabeled for ß-tubulin type III (monoclonal anti ßtubIII, 1:20,000) using a red fluorescent secondary antibody (goat anti mouse-alexaFluor568, Invitrogen, Germany, 1:250) and DAPI (0.1 µg/ml) as nuclear counterstain. Cells were examined on a Zeiss Axiovert 200 inverted microscope equipped with a CoolSnap camera (Photometrics, Tucson, AZ, USA) and MetaMorph software (Molecular Devices, Sunnyvale, CA, USA), or a Zeiss Axiocam50 mono and Zeiss ZENlite 2.6 blue edition software. Two photographs/well at × 20 magnification were taken. Lengths of all neurites in the field of view were measured in ImageJ (http://imagej.nih.gov/ij) using the ‚segmented line ‘ tool (Fig. 1c) and divided by the number of ß-tubulin III-positive cells. As an endpoint-specific control, cells in six wells were subjected to the rho-kinase inhibitor, Y-27632 (50 µM) which increases neurite outgrowth to more than 125% without affecting general viability. As a second endpoint-specific control, cells are subjected to cytochalasin D (100 nM), which reduces outgrowth to less than 25% as compared to control (see online supplementary material, Fig. 1a). Cell cultures that did not meet these criteria were discarded.

### Neuronal Differentiation Assay

As established in our previous study (Stern et al. 2014), cells were seeded into 96-well plates (Corning Costar, Kaiserslautern, Germany) at 80,000 cells per well and incubated in DMEM/F12 with 10 µM retinoic acid (RA) for 4 days (one media change). Cells were incubated for further 5 days (one media change) in media containing 10 µM RA and up to seven concentrations of a test compound. On day 9 (5-day exposition), cell viability was measured using a resazurin reduction assay (Trinova Biochem, Giessen, Germany). Subsequently, cells were fixed for 15 min in PFA and immunolabeled for ß-tubulin type III as above (Fig. 1a). For control purposes, photographs were taken at × 20 magnification as above. Neuronal differentiation was determined by measuring red fluorescence (excitation 568 nm, emission 603 nm) in a multiwell-plate reader (Infinite 200, TECAN, Germany). As a confirmation of the viability data, cell number was also quantified by measuring DAPI fluorescence (excitation 350 nm, emission 488 nm). As an endpoint-specific control, cells in six wells were subjected to sodium valproate (333 µM) which reduces neuronal differentiation to less than 50% while retaining general viability above 85% (Stern et al. 2014). Cell cultures that did not meet these criteria were discarded.

### Cell Migration Assay

As established before (Stern et al. 2014), NT2 precursor cells were seeded in 96-mm bacteriological grade Petri dishes (Greiner, Hamburg, Germany) at a density of 4 × 10^6^–5 × 10^6^ cells per dish. Within 24 h, cells formed free-floating spherical aggregates of 300–800 µm diameter. On the first day, 10 ml of culture medium was added to each Petri dish. On the next days, medium with 10 μM retinoic acid (RA) was added and changed every 2–3 days by transferring the cell suspension to centrifuge tubes and centrifuge at 200 × *g* for 7 min. After 9 days in culture, aggregates were gently resuspended using a Pasteur pipette, and seeded at 60,000 cells per well into black 96-well plates with flat transparent bottom (Nunc). Plates were lined with poly-D-lysine and laminin (10 µg/ml each), equipped with silicone stoppers from the Oris Cell Migration Assay (AMS Biotechnology, Abingdon, UK). Cells were allowed to adhere overnight. On the next day, stoppers were pulled leaving a monolayer of cells with a circular hole of 2-mm diameter. After one washing step to remove non-adherent cells, cultures were exposed to up to seven different concentrations of a test compound in DMEM/F12/RA (six technical replicates per concentration) for 44 h followed by resazurin reduction viability assay (2 h). After one wash in PBS, cells were fixed for 15 min in PFA, washed twice in PBS-T and stained with DAPI (0.5 µg/ml) for 5 min, followed by two washes in PBS. A black 96-hole plastic mask leaving a central circular area of 2-mm diameter for each well was clipped to the bottom of the plate. This allows viewing only those cells that had migrated into the free areas left by the silicone stoppers during seeding (Fig. 1b). Migration was quantified by taking two photographs of each well and measuring the distance of the migration front from the black margin of the mask in using ImageJ. Occasional asymmetries caused by slight inaccuracies of silicone stopper placement were compensated by averaging measurements in four quadrants per well. As a null-migration reference, six wells per plate were incubated with 100 nM cytochalasin D, which completely inhibits cell motility without affecting viability within 44 h (Stern et al. 2014). Average null-migration values were subtracted from migrated distance before evaluation. As an endpoint-specific control, cells in six wells were subjected to the rho-kinase inhibitor, Y-27632 (50 µM) which increases precursor cell migration to more than 125% without affecting general viability. Cell cultures that did not meet these criteria were discarded.

### Statistics

Concentration–response relationships were displayed as mean ± S.E.M. of three independent experiments using different passages of NT2-cells, normalized to untreated controls. To determine IC50 values, 4-parameter logistic curves were fitted to the data using GraphPad Prism8. Differences between individual values were evaluated by 2-way ANOVA followed by Dunnett’s test for multiple comparisons.

## Results

### Neurite Outgrowth Assay

**Fig. 2:**
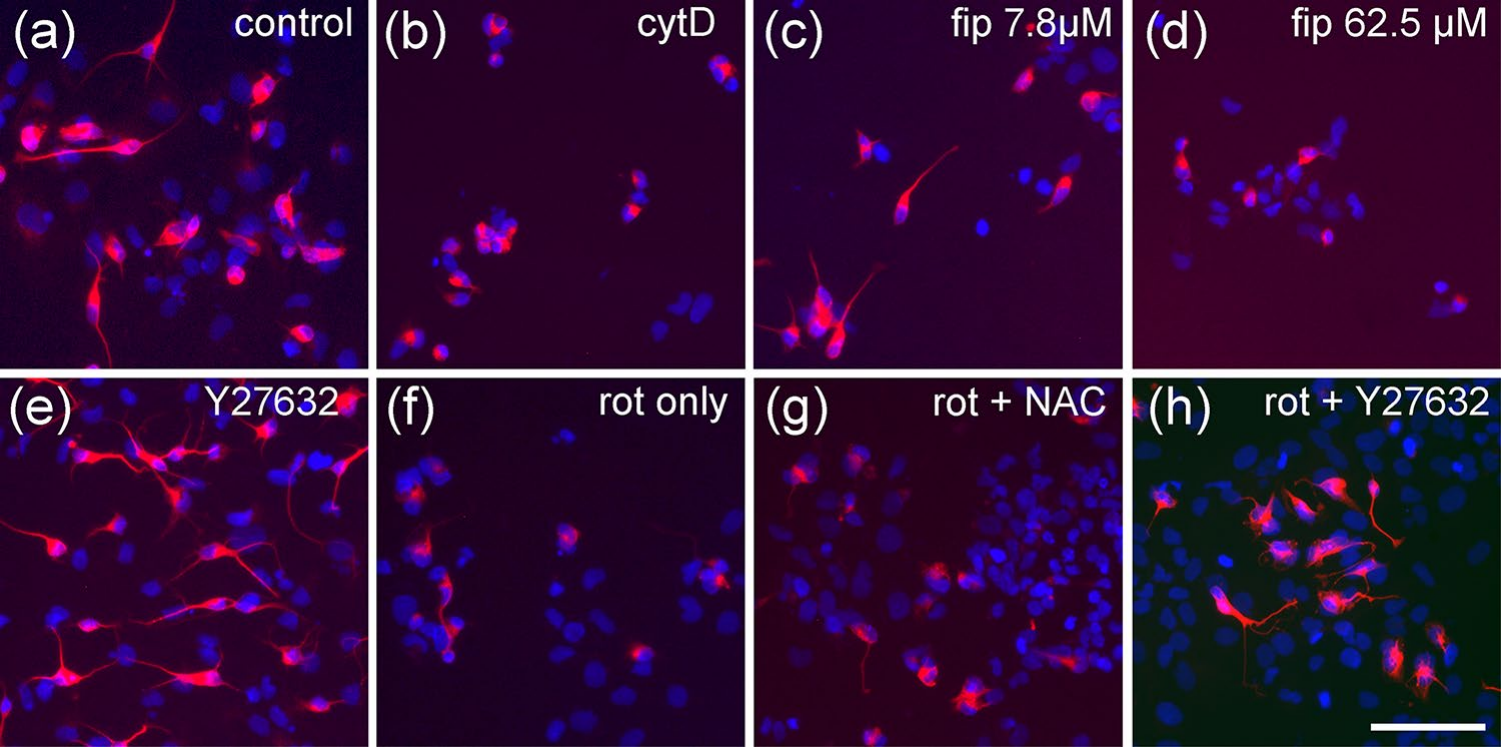
Neurite outgrowth assay. Examples are given for NT2 cells after 2 weeks of differentiation cultivated for 24 h in the presence of test compounds and labeled for neuron-specific ß-tubulin type III (red) and DAPI (blue). Cells without red fluorescence are still undifferentiated. Cells were subjected to (**a**) cell culture media only, (**b**) 100 µM cytochalasin D (cytD), (**c**) 7.8 µM fipronil (fip), (**d**) 62.5 µM fipronil, (**e**) 50 µM Y-27632, (**f**) 2.4 µM rotenone (rot only), (**g**) 2.4 µM rotenone plus 1.111 mM n-acetyl cysteine (rot + NAC), (**h**) 2.4 µM rotenone plus 50 µM Y-27632 (rot + Y27632). Scale bar: 100 µM

**Fig. 3:**
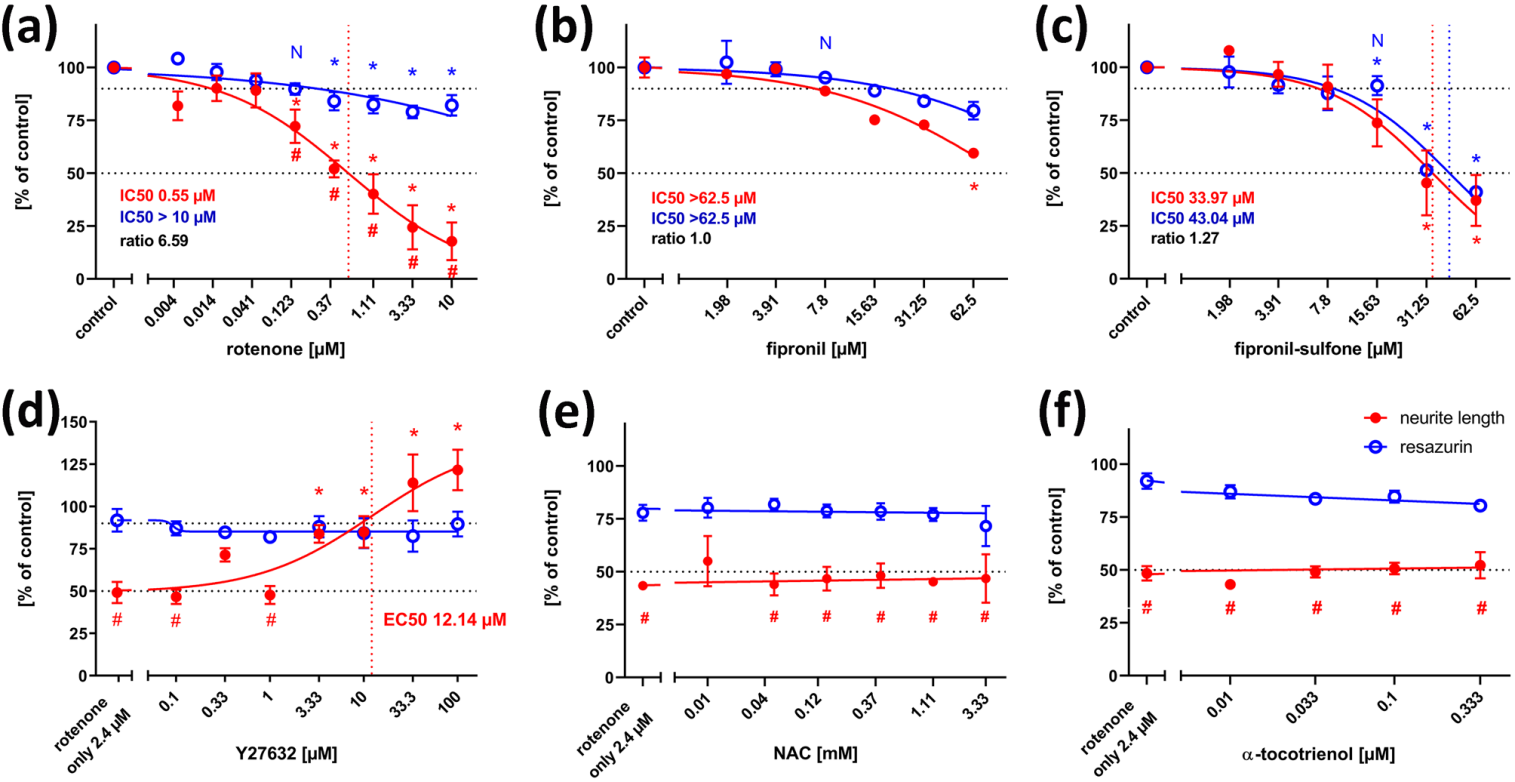
Neurite outgrowth assay, concentration–response curves. Each value is the average ± S.E.M. of three independent experiments normalized to the solvent control (0.25% DMSO). Hollow blue circles: general cytotoxicity (resazurin), filled red circles: total neurite length/neuron. Asterisks (*) indicate significant differences (at least *p* < 0.05) from controls, (#) indicate significant differences between viability and neurite length at that concentration. (N) indicates the highest non-cytotoxic concentration (viability > 90%, dotted horizontal line). **a** Rotenone reduced neurite outgrowth already at 123 nM, whereas general viability was still at 80% of control at the highest tested concentration (10 µM). **b** Fipronil reduced both viability and neurite outgrowth, but values below 50% were not reached at the highest concentration tested. **c** Fipronil sulfone reduced both viability and neurite outgrowth at the same concentrations. **d** The inhibitory effect of 2.4 µM rotenone on neurite outgrowth was compensated in a dose-dependent manner by the rho kinase inhibitor, Y-27632, with an EC50 of 12.14 µM. **e** The inhibitory effect of 2.4 µM rotenone on neurite outgrowth could not be alleviated by the antioxidant, n-acteyl cysteine (NAC). **f** The inhibitory effect of 2.4 µM rotenone on neurite outgrowth could not be alleviated by a second antioxidant, α-tocotrienol

As established before (Roloff et al. 2015), NT2 cells treated for 2 weeks with 10 µM retinoic acid in non-adherent dishes, and subsequently plated on poly-D-lysine, contain ~ 20% postmitotic, ß-tubulin type III-positive neurons a large portion of which readily grow neurites within 24 h (Figs. 1c, 2a). Neurite outgrowth could be enhanced by application of ROCK inhibitors like Y-27632 (Roloff et al. 2015, Fig. 2e). Neurite outgrowth is reduced by cytoskeletal inhibitors like cytochalasin D or colchicine in a dose-dependent manner (Fig. 1b, supplementary data Fig. 1). When treated with high concentrations of rotenone (2.4 µM), neurite outgrowth was strongly inhibited (Fig. 2f). Application of rotenone over a large range of concentrations (4 nM to 10 µM) revealed a concentration-dependent inhibition of neurite outgrowth with an IC50 of 0.55 µM (Fig. 3a). General cell viability was also significantly reduced (as compared to solvent controls) at higher rotenone concentrations but remained close to 80% even at the highest tested concentration. Thus, according to Krug et al. (2013), the IC50 for general toxicity was assumed as the highest tested concentration (10 µM), for calculatory purposes. At 123 nM rotenone, the highest non-cytotoxic concentration (viability > 90%), neurite outgrowth was already significantly reduced to less than 75%, indicating a specific DNT effect of rotenone on this endpoint. Comparing the IC50 values for neurite outgrowth and general cytotoxicity resulted in a ratio of 6.59. Krug et al. (2013) assumed IC50 ratios above a threshold value of 4 as indicative for DNT on neurite outgrowth of LUHMES cells, defined by the average ratio + 3 × standard deviation of unspecific test compounds. We applied the same procedure for neurite outgrowth of NT2 neurons using five different unspecific compounds and arrived at a threshold value of 2.02 (see Table 1, online supplementary material). In any case, the IC50 ratio of 6.59 for rotenone on NT2 neurite outgrowth is strongly indicative for a specific DNT effect of this compound. Since two alternative modes of action are discussed for rotenone, depending on ROS, or independently of ROS acting on the cytoskeleton via the rho-kinase pathway (Bisbal et al. 2018), we tested both alternatives for rotenone induced NT2 neurite outgrowth reduction. Application of the ROCK inhibitor, Y-27632, could alleviate the inhibitory effect of 2.4 µM rotenone on neurite outgrowth (Fig. 2h) in a dose-dependent manner (Fig. 3d). On the other hand, antioxidants like n-acetyl-cysteine (NAC, Fig. 3e) or α-tocotrienol (Fig. 3f) could not change rotenone-induced NT2 neurite outgrowth inhibition at any concentration.

Application of 62.5 µM fipronil also inhibited neurite outgrowth of NT2 neurons (Fig. 2c). However, concentration–response relations (Fig. 3b) revealed concurrent reductions of fipronil on general viability and neurite outgrowth, both of which did not fall below 50% at the highest testable concentration, resulting in an IC50 ratio of 1. Thus, again confirming the results by Krug et al. (2013), no specific DNT potential of fipronil on neurite outgrowth could be detected. Application of the main metabolic derivative of fipronil, fipronil sulfone, revealed a stronger inhibitory effect on both general viability and neurite outgrowth, resulting in IC50 values of 43.04 µM and 33.97 µM, respectively (Fig. 3c). However, the IC50 ratio of 1.27 failed to reach the threshold of 2.02, and significant differences between general cytotoxicity and inhibition of neurite growth could not be detected at any concentration. Thus, there appears to be no specific DNT potential on NT2 neurite outgrowth of fipronil sulfone.

### Differentiation Assay

**Fig. 4:**
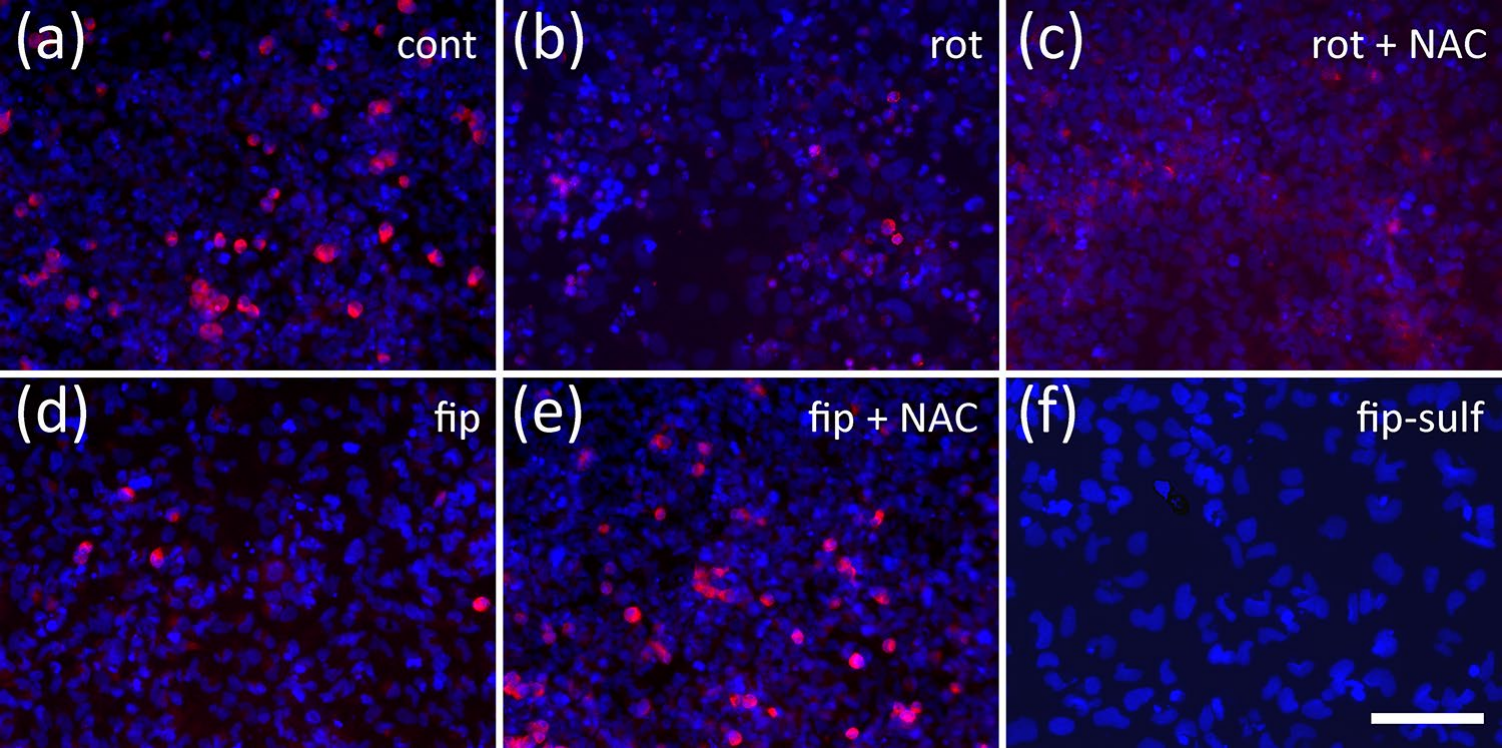
Neuronal differentiation assay. NT2 cells immunolabeled for ß-tubulin III, counterstained with DAPI after 9 days of differentiation, during last 5 days exposed to (**a**) DMSO only, (**b**) 11 nM rotenone, (**c**) 11 nM rotenone + 370 µM NAC, (**d**) 15 µM fipronil, (**e**) 15 µM fipronil + 1.111 mM NAC, (**f**) 15 µM fipronil sulfone. Scale bar 100 µm

**Fig. 5:**
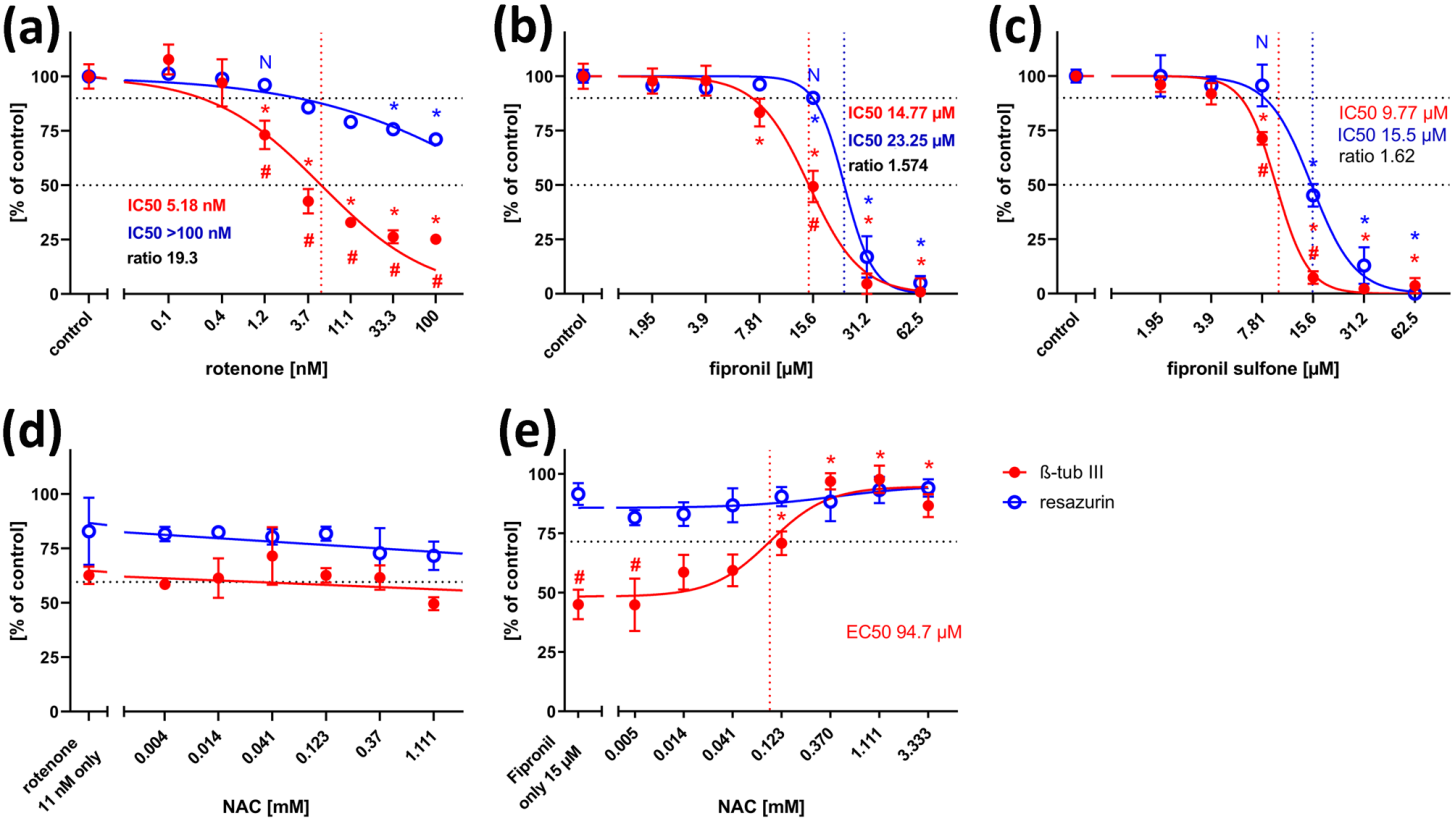
Neuronal differentiation assay, concentration–response curves. Each value is the average ± S.E.M. of three independent experiments normalized to the solvent control. Hollow blue circles: general cytotoxicity (resazurin), filled red circles: neuron-specific immunofluorescence of ß-tubulin type III after 4 days in cell culture media only, followed by 5 days of test compound exposure. Asterisks (*) indicate significant differences (at least *p* < 0.05) from solvent controls (**a**–**c**), exposure to rotenone only (**d**) or fipronil only (**e**), (#) indicate significant differences between viability and ß-tubulin III expression at that concentration. (N) indicates the highest non-cytotoxic concentration (viability > 90%, dotted horizontal line). (**a**) Rotenone reduced neuronal differentiation already at 1.2 nM, whereas general viability was still at 72% at the highest tested concentration. (**b**) Fipronil reduced both viability and neuronal differentiation in a dose-dependent manner. At 15.6 µM fipronil, differentiation was reduced to less than 50% and differed significantly from general viability (90%) at this concentration. (**c**) Fipronil sulfone reduced both viability and neuronal differentiation in a dose-dependent manner. At 7.81 µM, differentiation was significantly reduced to 70%, whereas general viability was still close to 100% at this concentration. (**d**) The inhibitory effect of 11 nM rotenone on neuronal differentiation could not be alleviated by the antioxidant, NAC, at any tested concentration. (**e**) The inhibitory effect of 15 µM fipronil was alleviated by the antioxidant, NAC, in a dose-dependent manner (EC50 94.7 µM)

In a previous study, we have shown that a specific DNT potential of toxic compounds on differentiation of NT2 precursor cells into neurons can be quantified by measuring immunofluorescence of the neuronal cytoskeletal marker, ß-tubulin type III (Stern et al. 2014). When we applied rotenone to cultures of differentiating NT2 cells, we saw a reduction in the number of ß-tubulin III-positive cells (Fig. 4b), as compared to solvent controls (Fig. 4a). Concentration–response relationships of measured ß-tubulin III-immunofluorescence revealed a concentration-dependent reduction of neuronal differentiation with an IC50 of 5.18 nM (Fig. 5a). At the highest non-cytotoxic rotenone concentration, 1.2 nM, ß-tubulin III-expression was already significantly reduced to below 75%. General viability of the cell culture was also reduced at high rotenone concentrations, but did not fall below 50% even at the highest tested concentration (100 nM). The IC50 ratio between general and specific toxicity (19.3) was much higher than the detection threshold of 2.77 determined by comparing IC50 ratios of five DNT negative compounds (Table 1, supplementary material). Application of high concentrations of fipronil (Fig. 4d) or fipronil sulfone (Fig. 4f) also reduced the number of ß-tubulin III-positive cells. Comparing concentration–response curves for general cytotoxicity and inhibition of neuronal differentiation by fipronil revealed a significant difference at 15.6 µM fipronil (Fig. 5b). At this concentration, viability was still above 90%, whereas ß-tubulin III-immunofluorescence was reduced to less than 50%, indicating a DNT potential of fipronil on NT2 differentiation. On the other hand, IC50s for general and specific toxicity differed only by a factor of 1.57, which is below the threshold value of 2.77 and thus fails this more strict criterion for DNT of fipronil. Similarly, fipronil sulfone reduced both viability and differentiation in a concentration dependent manner (Fig. 5c). At 7.81 µM fipronil sulfone, neuronal differentiation was significantly reduced to 70%, whereas viability was still unaffected. Also at 15.6 µM, differentiation was significantly more reduced than general viability, thus revealing a DNT potential of fipronil sulfone on NT2 differentiation.

Co-application of the antioxidant, NAC, to differentiating NT2 cells treated with 15 µM fipronil resulted in a concentration-dependent rescue effect with an EC50 of 94.7 µM NAC (Fig. 5e). At concentrations of 370 µM NAC and higher, the inhibitory effect of fipronil on NT2 differentiation could be completely negated, indicating a strong role of ROS in fipronil toxicity onto this endpoint. In contrast, NAC application could not alleviate the inhibitory effect of rotenone on ß-tubulin III-immunofluorescence at any concentration (Fig. 5d), indicating a ROS-independent mode of toxicity of rotenone on neural differentiation here.

### Cell Migration Assay

**Fig. 6:**
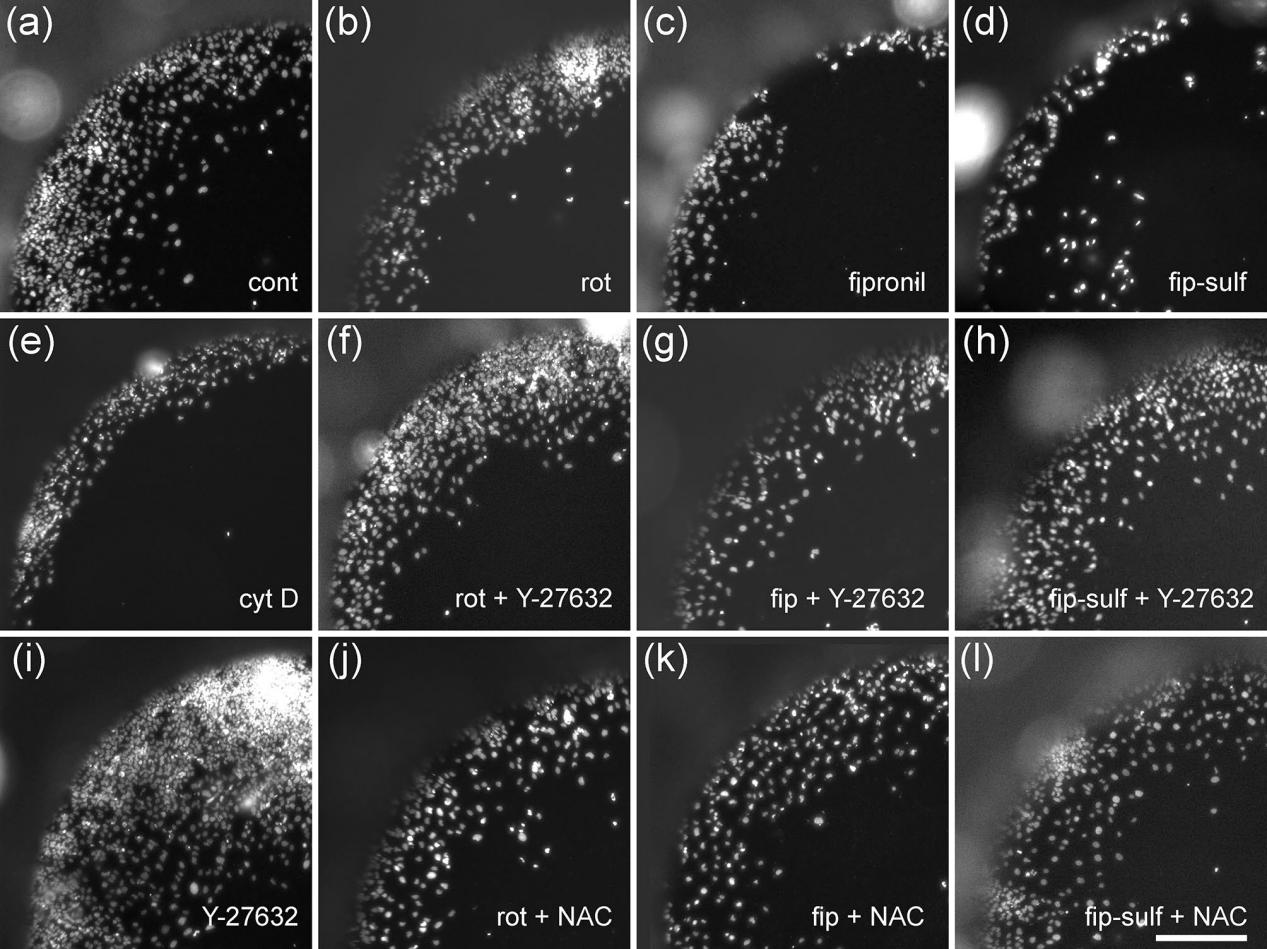
Migration assay. NT2 cells after 9 days of differentiation and 44 h exposed to test compounds, nuclei labeled with DAPI. Cells were exposed to (**a**) 0.25% DMSO only (cont) (**b**) 122 nM rotenone (rot), (**c**) 62.5 µM fipronil (fip), (**d**) 62.5 µM fipronil sulfone (fip-sulf), (**e**) 100 µM cytochalasin D (cyt D), (**f**) 122 nM rotenone plus 50 µM Y-27632, (**g**) 50 µM fipronil plus 50 µM Y-27632, (**h**) 15 µM fipronil sulfone plus 50 µM Y-27632, (**i**) 50 µM Y-27632, (**j**) 122 nM rotenone plus 1.111 mM n-acetyl-cysteine (NAC), (**k**) 50 µM fipronil plus 1.111 mM NAC, (**l**) 15 µM fipronil sulfone plus 1.111 mM NAC. Scale bar 400 µM

NT2 cells are also well suited for quantification of a specific DNT potential of toxic compounds on neuronal precursor migration (Stern et al. 2014). When cells were seeded in 96-well plates with circular silicon stoppers, cells migrated into the free space after removal of the stopper (Fig. 1b, Fig. 6). Within 44 h, the fastest cells migrated a distance of more than 400 µm under control conditions (Fig. 6a). Migration could be inhibited by cytoskeletal inhibitors such as cytochalasin D (Stern et al. 2014, Fig. 6e), and enhanced by the ROCK inhibitor, Y-27632 (Fig. 6i), allowing us to monitor both increase and reduction of cell migration upon treatment with chemicals.

**Fig. 7:**
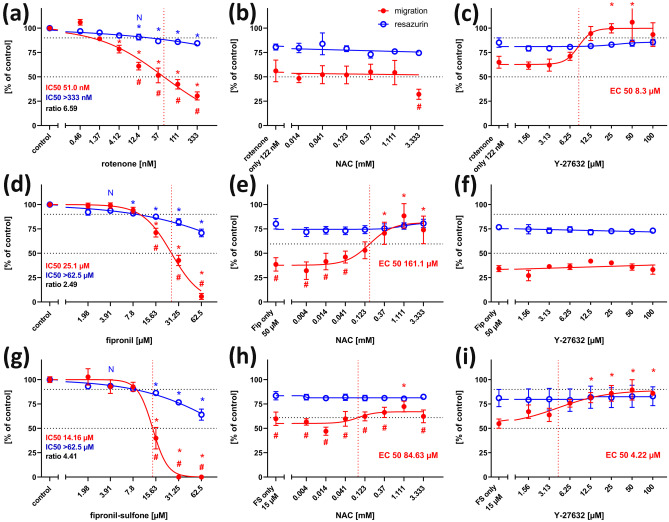
Migration assay, concentration–response curves. Each value is the average ± S.E.M. of three independent experiments normalized to the solvent control. Hollow blue circles: general cytotoxicity (resazurin), filled red circles: migration distance. Asterisks (*) indicate significant differences (at least *p* < 0.05) from controls, (#) indicate significant differences between viability and migration at that concentration. (N) indicates the highest non-cytotoxic concentration (viability > 90%, dotted horizontal line). (**a**) Rotenone reduced migration in a dose-dependent manner (IC50 51.0 nM), whereas general viability was reduced, but remained above 70% at the highest tested concentration. (**b**) The inhibitory effect of 122 nM rotenone could not be alleviated by the antioxidant, n-acetyl cysteine (NAC), at any concentration. (**c**) The inhibitory effect of 122 nM rotenone was alleviated by co-application of the rho kinase inhibitor, Y-27632, in a dose-dependent manner (EC50 8.3 µM). (**d**) Fipronil inhibited migration in a dose-dependent manner. At 15.63 µM fipronil and higher, migration was significantly reduced as compared to both migration of controls, and viability at the same concentration. (**e**) The inhibitory effect of 50 µM fipronil could be alleviated by the antioxidant, n-acetyl cysteine (NAC), in a dose-dependent manner (EC50 161.1 µM). **f** The inhibitory effect of 50 µM fipronil could not be alleviated by co-application of Y-27632 at any concentration. (**g**) Fipronil sulfone inhibited migration in a dose-dependent manner. At 15.63 µM fipronil and higher, migration was significantly reduced as compared to both migration of controls, and viability at the same concentration. (**h**) The inhibitory effect of 15 µM fipronil sulfone was slightly alleviated by the antioxidant, n-acetyl cysteine (NAC), in a dose-dependent manner (EC50 84.63 µM). At 1.111 µM NAC, cells migrated significantly further than under treatment of 15 µM fipronil sulfone (FS) alone. (**j**) The inhibitory effect of 15 µM fipronil sulfone was alleviated by co-application of the rho kinase inhibitor, Y-27632, in a dose-dependent manner (EC50 4.22 µM)

When treated with 122 nM rotenone, migration was strongly reduced (Fig. 6b). Migration was reduced in a concentration-dependent manner with an IC50 of 51 nM, whereas general viability was only slightly (but significantly) reduced even at the highest concentration tested (333 nM, Fig. 7a). A significant reduction of migration could already be observed at 4.12 nM rotenone. IC50 ratio between general cytotoxicity and inhibition of cell migration was 6.59, which is clearly above the threshold of 2.30 as calculated from IC50 ratios of DNT negative compounds (see supplementary material). Again, application of the ROCK-inhibitor, Y-27632 (Fig. 7c), but not the antioxidant, NAC (Fig. 7b), could reverse the inhibitory effect of rotenone on cell migration. Exposure to 62.5 µM fipronil strongly reduced NT2 cell migration (Fig. 6c). Concentration–response curves revealed a dose-dependent reduction of migration with an IC50 of 25.1 µM, whereas general viability was much less affected. Concentrations at or above 15.63 µM fipronil resulted in significant differences between viability and migration, and the IC50 ratio of 2.49 was (just) above our threshold criterion of 2.30, both indicating a DNT potential of fipronil on NT2 migration. Co-application of NAC to migrating NT2 cells exposed to 50 µM fipronil could significantly reduce this inhibition with an EC50 of 161.1 µM NAC (Fig. 7e), indicating the involvement of ROS in the DNT effect of fipronil on migration here. Application of Y-27632 could not alleviate fipronil-induced inhibition at any concentration (Fig. 7f). Fipronil sulfone also reduced cell migration (Fig. 6d, Fig. 7g) with an IC50 of 14.16 µM. At 15.63 µM fipronil sulfone, migration was significantly reduced to less than 40% as compared to viability, which remained at 85% at this concentration. The IC50 ratio of 4.41 clearly exceeds the threshold value of 2.30, indicating a strong DNT potential for fipronil sulfone on NT2 migration. In a rescue attempt with the antioxidant, NAC, the inhibitory effect of 15 µM fipronil sulfone was only slightly alleviated in a concentration-dependent manner. At 1.111 mM NAC, migration was significantly faster than under treatment of fipronil alone (Fig. 6l), indicating a possible partial involvement of ROS in fipronil sulfone toxicity on NT2 migration. In contrast, application of Y-27632 to migrating cells exposed to 15 µM fipronil sulfone strongly reduced its inhibitory effect with an EC50 of 4.22 µM, indicating that fipronil sulfone acts on NT2 precursor cell migration to a large extent in a ROS-independent manner.

## Discussion

In the present study, we investigated the effects of three insecticide compounds on three different developmental neurotoxic endpoints in the human NT2 cell line. Proliferating NT2 precursor cells terminally differentiate into postmitotic neurons upon treatment with a single morphogen, retinoic acid (Andrews et al. 1984; Pleasure et al. 1992). NT2 neurons express neuronal cytoskeletal proteins, including ß-tubulin type III, and a broad variety of different neurotransmitter phenotypes (Guillemain et al. 2000; Podrygailo et al. 2009). They acquire both presynaptic proteins (Podrygajlo et al. 2009) and synaptic vesicle release mechanisms (Tegenge et al. 2009) as well as postsynaptic currents (Podygajlo et al. 2010). NT2 neurons have been successfully grafted into mammals (Saporta et al. 2002) and human patients (Nelson et al. 2002). The suitability of NT2 cells for detecting alterations to early steps of neural development has been shown for various key events such as neural differentiation (Hill et al. 2008; Dahm et al. 2010; Stern et al. 2014), precursor cell migration (Tegenge and Bicker, 2009; Tegenge et al. 2011; Stern et al. 2014), and neurite outgrowth (Reuss and Asif 2014; Roloff et al. 2015).

In the present study, we have shown that the insecticide, rotenone, impaired all three tested DNT endpoints, neurite outgrowth, neuronal differentiation, and precursor cell migration in a dose-dependent manner and clearly separable from general cytotoxicity. These results are in line with the findings by Krug et al. (2013) and Ryan et al. (2016) with regard to neurite outgrowth and the effects on neuronal differentiation reported by Pamies et al. (2018) and Pistollato et al. (2017). On the other hand, in an in vivo setting, rotenone specifically inhibited directed neurite growth in an intact insect embryo (Bergmann et al. 2019; Bode et al. 2020). A negative effect of rotenone on cell migration out of neurospheres has been reported by Ishido and Suzuki (2010) with an IC50 of ca. 200 nM, but general cytotoxicity had not been tested in their study. Interestingly, IC50 values for rotenone differed by an order of magnitude between different endpoints, with differentiation being affected by the lowest concentrations, whereas neurite outgrowth being the least sensitive, and migration in between. This could simply reflect different durations of toxin exposition in the assays but might also hint to varying sensitivity to chemical disturbance during neural development. Various modes of action of rotenone on cells have been proposed. Since rotenone is a mitochondrial complex I inhibitor, the generation and detrimental intracellular effects of reactive oxygen species (ROS) could play an important role. Evidence for the involvement of ROS in rotenone neurotoxicity has been demonstrated, e.g., by Li et al. (2003), Pamies et al. (2018), and Pistollato et al. (2017). Alternatively, a more direct interaction with the cytoskeleton could also contribute to rotenone toxicity. Marshall and Himes (1978) have demonstrated that rotenone can directly inhibit microtubule polymerization in vitro. This mechanism is less likely to play a role here, since concentrations up to 5–20 µM are necessary for microtubule inhibition, whereas for the least sensitive endpoint, neurite outgrowth, IC50 is 0.55 µM in our experiments (Fig. 3a). However, rotenone can also act on the cytoskeleton via the rho A-ROCK pathway (Bisbal et al. 2018). The inhibitory effect of rotenone could be counteracted by inhibition of this pathway by Y-27632 for migration and neurite outgrowth in our experiments and also for neurite formation in LUHMES cells (Krug et al. 2013). In contrast, antioxidants like tocotrienol or NAC did not counteract the DNT effect of rotenone. Thus, at the low effective concentrations of rotenone, activation of the rho/ROCK pathway appears to have a much stronger effect than ROS in our system. How exactly rotenone exerts its effect on this pathway has yet to be elucidated. In the postnatal brain, dopaminergic neurons are particularly sensitive to rotenone exposure (Betarbet et al. 2000), and also in vitro developmental testing has been largely focused on cells with a dopaminergic context (Krug et al. 2013; Pamies et al. 2018). NT2 cells hardly develop into dopaminergic neurons, however (Podrygajlo et al. 2009), which supports the view that rotenone is a general developmental neurotoxicant rather than specifically attacking only dopaminergic cells.

The status of fipronil as a developmental neurotoxicant is much less clear. As an insecticide, fipronil is directed against insect GABAa receptors, to which it has a more than 100-fold higher affinity than to vertebrate GABA receptors (Hainzl et al. 1996). However, in the body, fipronil is quickly converted into its primary oxidation product, fipronil sulfone, which inhibits invertebrate GABA receptors much less selectively than invertebrate GABA receptors (Hainzl et al. 1996). In the zebrafish embryo, development of motor circuits is disturbed by fipronil at 0.7 µM, most likely by interaction of fipronil with glycin receptors (Stehr et al. 2006). At higher concentrations (~ 33 µM), fipronil inhibits zebrafish neurogenesis via reduction of gene expression important for neuron/glia differentiation (Park et al. 2020).

In our in vitro experiments on developing human neurons, we could test fipronil at concentrations up to 62.6 µM. We saw both reduction of neurite outgrowth and cell viability at this concentration, but no specific DNT effect of fipronil on neurite outgrowth could be separated, confirming the results of Krug et al. (2013) on LUHMES cells. Similar results were obtained for fipronil sulfone, which appeared to be slightly more toxic, but again, reduction of neurite outgrowth could not be separated from general cytotoxicity. Sidiropoulou et al. (2011) have demonstrated reduced outgrowth of axon-like processes from N2a neuroblastoma cells when treated with 1 µM fipronil. In SH-SY5Ycells, 25 µM fipronil reduced process outgrowth of vimentin-positive protrusions of incompletely differentiated cells, but not viability (Ruangjaroon et al. 2017). However, these processes or protrusions probably have little in common with fully determined ß-tubulin III-positive axons or dendrites. We can conclude that fipronil may interfere with parts of the cytoskeleton prior to terminal neuronal differentiation, but effects on genuine neurites cannot be separated from general cytotoxicity.

In contrast, migration of NT2 precursor cells was clearly and specifically inhibited by fipronil and fipronil sulfone in our experiments. Both the IC50 ratios are above the threshold (Table 2), and migration is already significantly reduced by near non-cytotoxic concentrations of fipronil (Fig. 7d) or fipronil sulfone (Fig. 7g). To the best of our knowledge, this is the first instance of a clear DNT effect of fipronil on human early CNS developmental processes shown in vitro. The situation is less clear for neuronal differentiation. Both fipronil and fipronil sulfone inhibited differentiation of NT2 precursors into ß-tubulin III-immunoreactive neurons at IC50 of 14.77 µM and 9.77 µM, respectively. However, general viability was also affected, and IC50 ratios were below the prediction threshold, indicating possible unspecific effects. On the other hand, at clearly non-cytotoxic concentrations (15.6 µM fipronil, 7.81 µM fipronil sulfone), neuronal differentiation was significantly different from both media control and from general viability at this concentration. These results indicate a potential specific DNT effect of fipronil and its derivative on human neuronal differentiation. This view is supported by the finding that fipronil can shift differentiation of rat neural epithelial stem cells from neurons to glia (Slotkin et al. 2016). In comparison, fipronil sulfone displayed stronger toxicity (lower IC50) on NT2 cells than fipronil for all three endpoints. This is in line with the results of Romero et al. (2016) in SH-SY5Y cells.

**Table 2:** Summary of the results

	Compound	IC50			Prediction model	DNT (IC50 ratio)	DNT (sign diff)	Rescue	
		unspec. EP	spec. EP	Ratio				Y-27632	NAC
Neurite outgrowth	Rotenone	> 10 µM	550.5 nM	18.16	2.02	Yes	Yes	Yes	no
	Fipronil	> 62.5 µM	> 62.5 µM	1.0		No	No	n.d	n.d
	Fipronil sulfone	43.04 µM	33.97 µM	1.27		No	No	n.d	n.d
Migration	Rotenone	> 333 nM	51.0 nM	6.53	2.30	Yes	Yes	Yes	no
	Fipronil	> 62,5 µM	25.1 µM	2.49		Yes	Yes	Yes	yes
	Fipronil sulfone	> 62.5 µM	14.16 µM	4.41		Yes	Yes	Yes	yes
Neuronaldifferentiation	Rotenone	> 100 nM	5.18 nM	19.3	2.77	Yes	Yes	No	no
	Fipronil	23.35 µM	14.77 µM	1.58		No	Yes	n.d	yes
	Fipronil sulfone	15,5 µM	9,77 µM	1.62		No	Yes	n.d	no

Threshold IC50 ratios for the prediction model are based on IC50 ratios + 3 × s.d. of five DNT-unspecific compounds (see online supplementary material Table 1) for each endpoint. DNT (IC50 ratio) is positive (green) when the IC50 ratio is larger than the threshold IC50 ratio. DNT (sign diff) is positive (green) when there is at least one concentration of the compound where the specific endpoint is significantly different from both control of the specific endpoint and the unspecific endpoint at that concentration. Rescue is positive (yes), when there is at least one concentration of the rescuing compound where the specific endpoint is significantly less affected by the inhibitory effect of the test compound than by the test compound alone. *n.d.* not detected

In the early stages of neuronal development assessed in this study, there is no synaptic transmission by GABA (or any other transmitter) yet. The first evidence for the establishment of synapses between fully matured NT2 neurons appear after additional 7 days in culture (Tegenge et al. 2009). Alternatively, oxidative stress is considered a major mechanism of fipronil (and fipronil sulfone) neurotoxicity based on the amelioration of fipronil toxicity by co-application of antioxidants like vitamin E derivatives or NAC (Ki et al. 2012; Romero et al. 2016). In our experiments, application of NAC could completely counteract the specific inhibition of NT2 migration by 50 µM fipronil (Fig. 7e). Interestingly, co-application of NAC could only slightly counteract the inhibition of migration by 15 µM fipronil sulfone (Fig. 7h). The lower efficacy of antioxidants on detrimental effects of fipronil sulfone, as compared to fipronil, has also been observed by Romero et al. (2016) in SH-SY5Y cells. Since ROS are generated mainly by incomplete oxidation of fipronil through CYP450 enzymes (Wang et al. 2016), the already oxidized compound fipronil sulfone might act in a different way. The application of the ROCK inhibitor Y-27632 could counteract inhibition of NT2 precursor cell migration by fipronil sulfone, indicating, again, the involvement of the rho/ROCK pathway here. It is unlikely that the bioconversion of fipronil into fipronil sulfone by NT2 cells in culture contributed much to the toxicity in our experiments, because the low number of cells compared to the large volume of incubation media preclude the generation of the high concentrations of fipronil sulfone necessary for cytotoxic action.

Generally, IC50 values for both fipronil and its oxidation product are quite high, in the micromolar range, as compared to rotenone (IC50 in the nanomolar range). Blood fipronil sulfone concentrations in the average human urban population of South Korea range in the low nanomolar range (Kim et al. 2019). This keeps direct DNT effects on early neural developmental events as in the present in vitro study unlikely, even though up to 1.1 nM fipronil sulfone was measured in umbilical cord blood, and this was correlated with reduced Apgar scores in infants (Kim et al. 2019). In a study on workers with occupational fipronil exposure, blood fipronil sulfone concentrations up to 0.1 µM were reached (Herin et al. 2011), which is still far below toxic concentrations in our study. However, a study on patients with strong acute intoxication because of voluntary or accidental oral insecticide uptake revealed blood concentrations up to 8.6 µM (Mohammed et al. 2004), which is close to the IC50 of 9.77 µM for neuronal differentiation determined in the present study.

## Conclusion

We demonstrate a direct, specific DNT effect of fipronil and its primary oxidation product, fipronil sulfone, onto developing human neural precursor cells. Migration of neural precursor cells is more clearly affected than neuronal differentiation, whereas inhibition of neurite outgrowth cannot be separated from general cytotoxicity. However, since the disturbance of even a single neurodevelopmental key event will cause an adverse neurodevelopmental outcome (Baumann et al. 2016), fipronil should be considered DNT. In this light, the suggestion of fipronil as a clear DNT negative model substance for the development of in vitro assays (Aschner et al. 2017) should be reconsidered. In practice, moderate fipronil exposure by normal food consumption even of a few “poison eggs” is unlikely to exert adverse outcome onto infant brain development through interference of fipronil with early developmental events such as migration, differentiation, or neurite outgrowth in humans. Nevertheless, the role of metabolic products of potentially harmful chemicals should be given more attention in in vitro assays.

## Supplementary Information

Below is the link to the electronic supplementary material.

Supplementary file1 (PDF 836 KB)

## Data Availability

Data used in this study are available on reasonable request.
