# Supplementary material for: Developmental Neurotoxicity of Fipronil and Rotenone on a Human Neuronal In Vitro Test System
Source: Neurotox Res. 2021 Apr 19;39(4):1189–202. doi: 10.1007/s12640-021-00364-8 (PMC8275550; doi:10.1007/s12640-021-00364-8)
Supplement: Supplementary file 1 — Supplementary file1 (PDF 836 KB) [file 12640_2021_364_MOESM1_ESM.pdf]

Developmental neurotoxicity of fipronil and rotenone on a human neuronal in vitro test system

Online supplementary material

Anne Schmitz, Silke Dempewolf, Saime Tan, Gerd Bicker, and Michael Stern

University of Veterinary Medicine Hannover, Institute of Physiology and Cell Biology,

Bischofsholer Damm 15/102, D-30173 Hannover, Germany

Running title: developmental toxicity of fipronil in vitro

Corresponding author: Michael Stern

University of Veterinary Medicine Hannover

Institute of Physiology and Cell Biology

Bischofsholer Damm 15/102

D-30173 Hannover, Germany

Email: Michael.stern@tiho-hannover.de

Phone: +49 511 856 7767

Fax: +49 511 856 7687

(a)

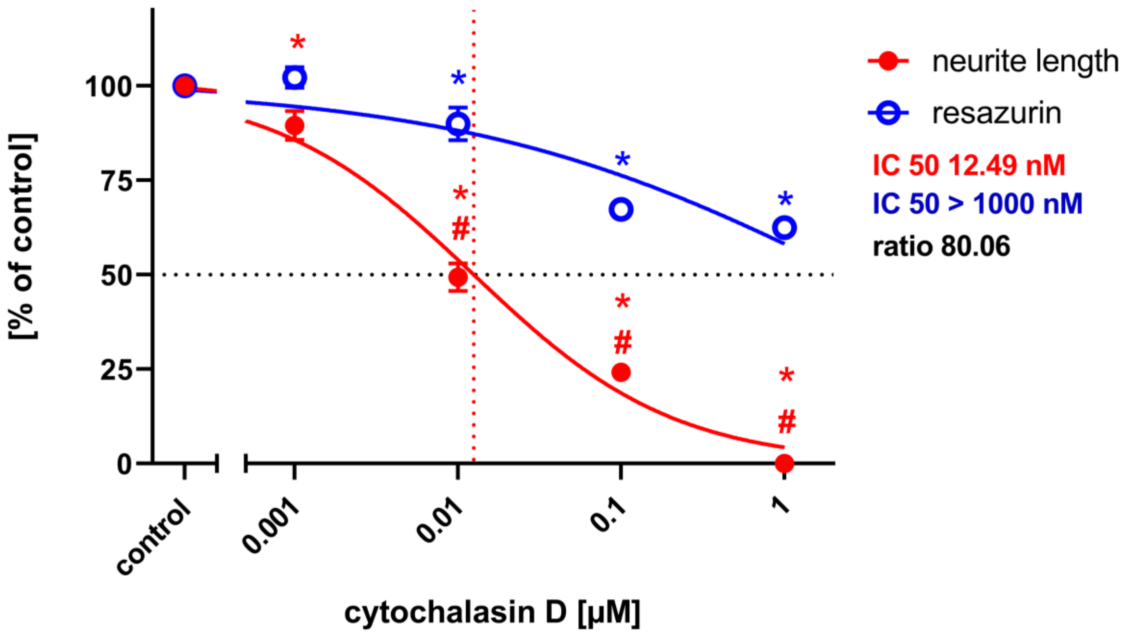

(b)

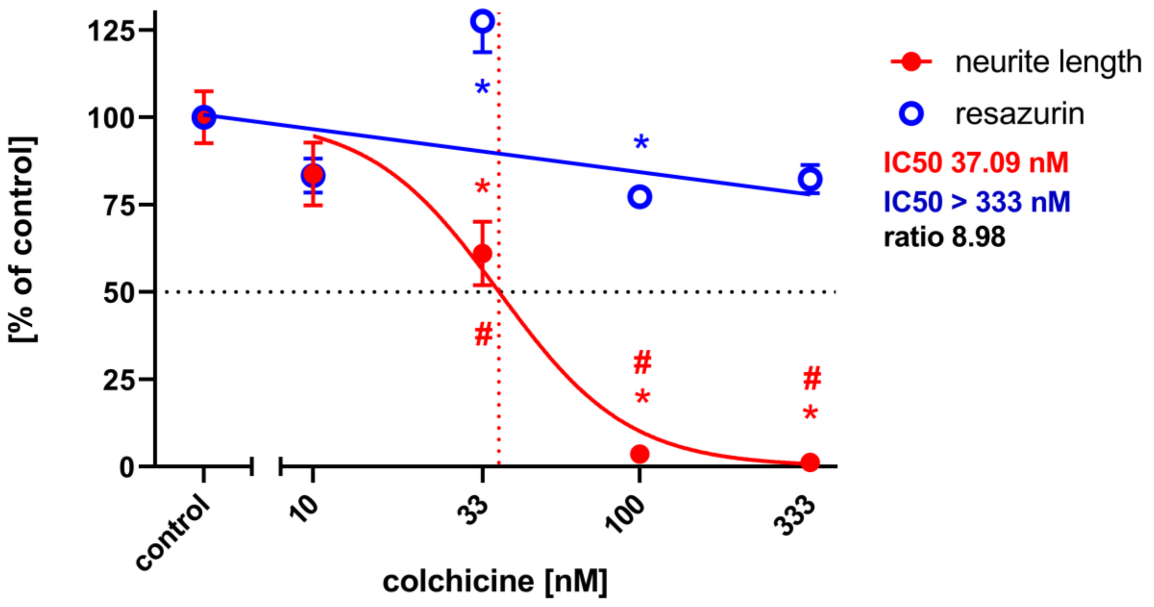

Online supplementary material Fig. 1 Neurite outgrowth assay, concentration-response curves of endpoint specific control compounds: (**a**) **Cytochalasin D** specifically inhibits neurite outgrowth. (**b**) **Colchicine** specifically inhibits neurite outgrowth. Each value is the average ± S.E.M. of three independent experiments normalized to the solvent control (0.25% DMSO). Hollow blue circles: general cytotoxicity (resazurin), filled red circles: total neurite length/neuron. Asterisks (*) indicate significant differences (at least $p<0.05$) from controls, (#) indicate significant differences between viability and neurite length at that concentration.

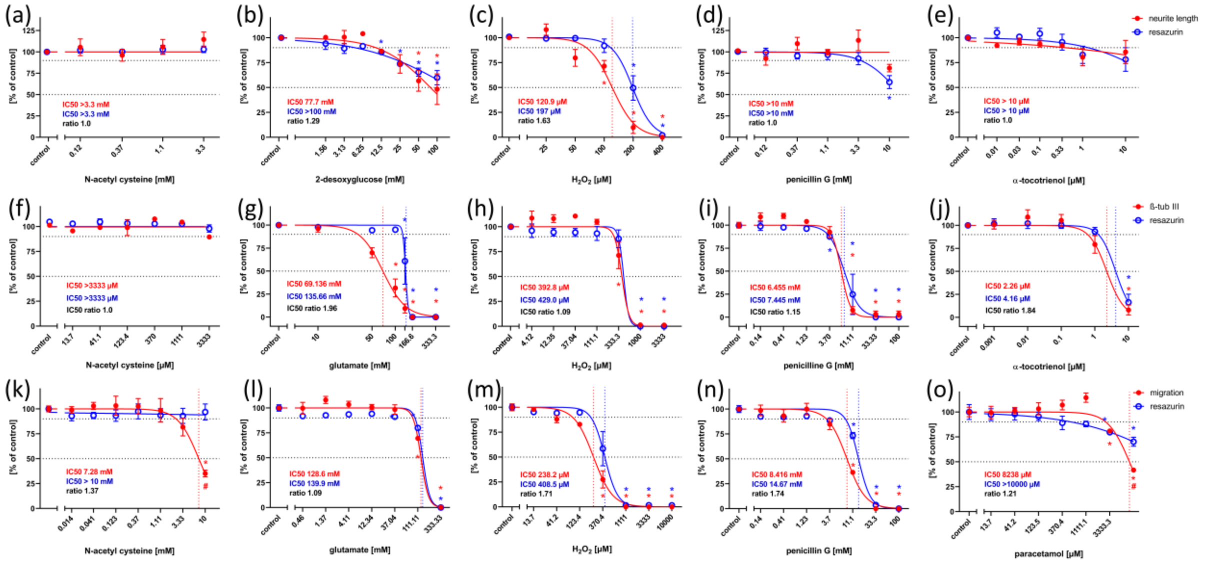

Online supplementary material Fig. 2 Concentration-response curves of five unspecifically toxic compounds for all three endpoints: **neurite outgrowth (a-e), neuronal differentiation (f-j), neural precursor cell migration (k-o).** Each value is the average ± S.E.M. of three independent experiments normalized to the solvent control (0.25% DMSO). Hollow blue circles: general cytotoxicity (resazurin), filled red circles: specific endpoint. Asterisks (*) indicate significant differences (at least p<0.05) from controls, (#) indicate significant differences between viability and neurite length at that concentration.

	**neurite outgrowth**		**migration**		**neuronal differentiation**	
	**compound**	**ratio**	**compound**	**ratio**	**compound**	**ratio**
	TOC	1,00	AAP*	1,21	PG*	1,15
	NAC	1,00	PG*	1,74	Glu*	1,96
	PenG	1,00	Glu*	1,09	NAC	1,00
	H_2O_2	1,63	NAC	1,37	TOC	1,84
	2DG	1,29	H_2O_2	1,71	H_2O_2	1,09
mean		1,18		1,42		1,41
s.d.		0,28		0,29		0,45
3x s.d.		0,84		0,88		1,36
mean + 3x s.d.		**2,02**		**2,30**		**2,77**
*: data taken from Stern et al. (2014) Arch. Toxicol. 88: 127–136						

Online supplementary material Table 1 Unspecific compounds: IC50 ratios between general cytotoxicity measured by resazurin reduction assay and specific endpoints. Each value is the ratio IC50 unspecific/IC50 specific derived from concentration-dependence curves (Online supplementary material Fig. 2) from three independent experiments on NT2 cultures as described in the materials and methods section. The mean + 3 standard deviations (s.d.) of five different unspecific compounds are used as threshold IC50 ratios. According to Krug et al. (2013), we consider a chemical compound DNT positive for any given endpoint, when the IC50 ratio exceeds this threshold. 2DG: 2-desoxy glucose, AAP: acetaminophen (paracetamol), Glu: sodium glutamate, H_2O_2: hydrogen peroxide, NAC: n-acetyl cysteine, PG: penicillin G, TOC: α-tocotrienol.

References:

Krug AK, Balmer NV, Matt F, Schonenberger F, Merhof D, Leist M (2013) Evaluation of a human neurite growth assay as specific screen for developmental neurotoxicants Arch Toxicol 87:2215-2231 doi:10.1007/s00204-013-1072-y

Stern M, Gierse A, Tan S, Bicker G (2014) Human Ntera2 cells as a predictive in vitro test system for developmental neurotoxicity Arch Toxicol 88:127-136 doi:10.1007/s00204-013-1098-1
